# Supplementary figures and images for: Hepatotropic Properties of SARS-CoV-2—Preliminary Results of Cross-Sectional Observational Study from the First Wave COVID-19 Pandemic
Source: J Clin Med. 2021 Feb 9;10(4):672. doi: 10.3390/jcm10040672 (PMC7916209; doi:10.3390/jcm10040672)

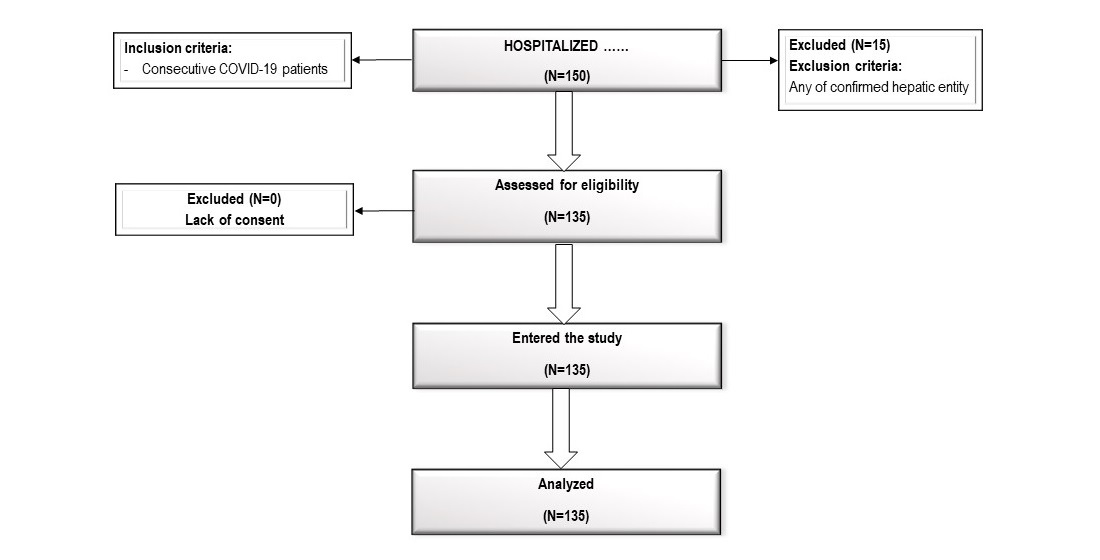

Supplement: Supplementary file 1 [file jcm-10-00672-s001.zip › jcm-1050189-supplementary.jpg]
